# The roles of TAF1 in neuroscience and beyond

**DOI:** 10.1098/rsos.240790

**Published:** 2024-09-25

**Authors:** Elisa M. Crombie, Karen Cleverley, H. T. Marc Timmers, Elizabeth M. C. Fisher

**Affiliations:** ^1^ Department of Neuromuscular Diseases, UCL Institute of Neurology, University College London, London WC1N 3BG, UK; ^2^ German Cancer Research Center (DKFZ), Im Neuenheimer Feld 280, Heidelberg 69120, Germany; ^3^ German Cancer Consortium (DKTK), partner site Freiburg, a partnership between the DKFZ, Germany; ^4^ Department of Urology, Medical Center-University of Freiburg, Breisacher Straße 66, Freiburg, 79106, Germany

**Keywords:** TATA box binding-protein (TBP)-associated factor 1, TAF1, X-linked dystonia–parkinsonism, X-linked intellectual disability, TFIID, RNA polymerase II

## Abstract

The transcriptional machinery is essential for gene expression and regulation; dysregulation of transcription can result in a range of pathologies, including neurodegeneration, cancer, developmental disorders and cardiovascular disease. A key component of RNA polymerase II-mediated transcription is the basal transcription factor IID, which is formed of the TATA box-binding protein (TBP) and 14 TBP-associated factors (TAFs), the largest of which is the TAF1 protein, encoded on the X chromosome (Xq13.1). *TAF1* is dysregulated in X-linked dystonia–parkinsonism and congenital mutations in the gene are causative for neurodevelopmental phenotypes; TAF1 dysfunction is also associated with cardiac anomalies and cancer. However, how TAF1 contributes to pathology is unclear. Here, we highlight the key aspects of the *TAF1* gene and protein function that may link transcriptional regulation with disorders of development, growth and adult-onset disorders of motor impairment. We highlight the need to experimentally investigate the full range of TAF1 messenger RNA variants and protein isoforms in human and mouse to aid our understanding of TAF1 biology. Furthermore, the X-linked nature of *TAF1*-related diseases adds complexity to understanding phenotypes. Overall, we shed light on the aspects of TAF1 biology that may contribute to disease and areas that could be addressed for future research and targeted therapeutics.

## 1. Introduction

TATA box binding-protein (TBP)-associated factor 1 (TAF1) plays a key role in the initiation of RNA polymerase II (pol II)-dependent transcription. Human TAF1 is encoded by a large gene located on the X chromosome (Xq13.1), with multiple splice variants of mostly unexplored function and tissue specificity. TAF1 is best known for being part of a protein complex with TBP and other TAFs, forming the basal transcription factor IID (TFIID), which is an essential component of the RNA pol II initiation complex [1]. Similarly to all other TAFs, TAF1 is a highly conserved protein, which has undergone only limited changes throughout eukaryotic evolution [2]. This testifies to the central role TAF1 plays in eukaryotic transcription regulation.

Since the *TAF1* gene is located on the q arm of the human X chromosome, genetic conditions affecting TAF1 function are X-linked and predominantly affect males. Missense mutations in the *TAF1* gene can cause X-linked syndromic mental retardation−33 (MRXS33) also known as X-linked intellectual disability (XLID), which presents with heterogeneous clinical features [3]. Moreover, *TAF1* is dysregulated in X-linked dystonia–parkinsonism (XDP), a progressive neurodegenerative condition, arising from the insertion of a retrotransposon into intron 32 of the gene [4–6]. XDP brains have striatal atrophy and patients manifest late-onset motor impairment similar to that in Huntington’s disease (HD) [7,8], in which reduced TAF1 protein expression has been reported [9]. However, why dysfunction of ubiquitously expressed *TAF1* predominantly affects the brain and leads to neurological disorders when mutated is unclear. Much remains to be discovered about this large, fundamentally important protein including the roles of its multiple splicing isoforms.

### 1.1. TAF1 protein function

**Figure 1.**
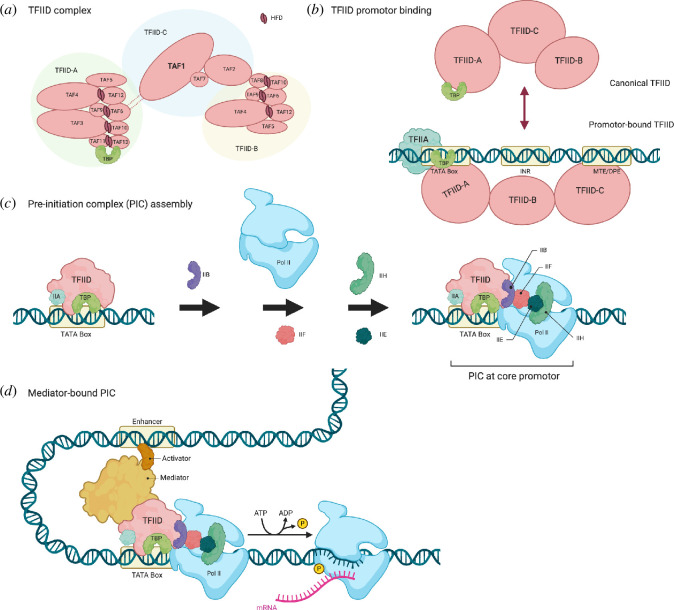
Transcription initiation complex assembly. (*a*) TFIID complex. TFIID is organized into three lobes: lobes A, B and C (TFIID-A, -B and -C). Certain TAFs are present in both lobes A and B and several heterodimerize through their histone-folding domains (HFDs). The cryo-electron microscopy (cryoEM)-visible parts of TAF1 locate/within TFIID-C i.e. within the C lobe. (*b*) TFIID exists in two major conformations: ‘canonical’ or ‘promoter-bound’ TFIID. Canonical TFIID exists in a relatively compact conformation, whereas promoter-bound TFIID binds to the TATA box via TBP together with TFIIA, stabilizing the complex at the core promoter. Aside from the TATA box, eukaryotic core promoters comprise multiple elements including initiator (INR), motive ten element (MTE) and downstream core promoter element (DPE) [11]; for (*c*) and (*d*), only the TATA box is shown for simplicity. TFIID structure and conformational changes are based on cryoEM data [10,12] and are described in Malik & Roeder [13]. (*c*) Pre-initiation complex (PIC) assembly. TFIID recognizes the core promoter of a gene to be transcribed, with TBP and TFIIA, then further recruits TFIIB, RNA polymerase II (pol II), TFIIF, TFIIH and TFIIE to assemble the PIC. (*d*) Mediator-bound PIC. Basal (a.k.a. general) transcription factors, pol II, and the coactivator mediator are recruited to the PIC [11]. Mediator facilitates interactions between the TFIID and transcriptional activators. Current models are reviewed in detail in Malik & Roeder [13]. Size of DNA and proteins not to scale. TAF, TBP-associated factor; TBP, TATA box-binding protein; (TF)IIA, B, D, E, F, H, basal transcription factor 2A, B, D, E, F, H. Created with BioRender.com.

The canonical TAF1 protein comprises 1873 amino acids with a molecular mass of 250 kDa. TAF1 is the largest component of the human TFIID complex, which acts as a basal transcription factor for all pol II-mediated transcription in eukaryotes. As part of TFIID, TAF1 acts together with TBP and other TBP-associated factors (18−140 kDa), including TAF2, TAF3, TAF4, TAF4B, TAF5, TAF6, TAF7, TAF8, TAF9, TAF9B, TAF10, TAF11, TAF12 and TAF13 ([10]; figure 1*a*).

TFIID is organized into three structural lobes A, B and C. TBP is bound in an inhibited form to the A lobe (TFIID-A) [14]. Nine TAFs heterodimerize at an interface of their histone-fold domains, specifically TAF4–TAF12, TAF6–TAF9, TAF3–TAF10, TAF8–TAF10 and TAF11–TAF13 [15]. The core TFIID TAFs (TAF4/TAF4B, TAF5, TAF6, TAF9/TAF9B and TAF12) are present in two copies across A and B lobes [14]. TAF1 is the core scaffold protein of the TFIID complex and is located within the C lobe (TFIID-C) [14]. TAF1 is involved in nucleating complex assembly and, as part of TFIID, is shown to form a promoter DNA-binding subcomplex, together with TAF7 and TAF2 [10]. A trimeric TAF2−8–10 complex also forms in the cytoplasm, where TAF8 promotes TAF2 incorporation into TFIID [16]. As the first basal transcription factor, TFIID recognizes the core promoter irrespective of a TATA box (figure 1*b*), promoting the assembly of the pre-initiation complex (PIC), which comprises pol II and other basal transcription factors (TFIIA, TFIIB, TFIID, TFIIE, TFIIF and TFIIH) (figure 1*c*). It has been shown by cryoelectron microscopy (cryoEM) that PIC complexes assemble on three types of core promotors: TATA-less, TATA-only and TATA-DBE (consisting of an upstream TATA box and downstream TFIID-binding elements [DBEs]). There are two tracks of promotor assembly where the PIC is directly deposited on TATA-less and TATA-only promotors compared with the stepwise approach for TATA-DBE [10]. Further detail on TFIID complex assembly has been described in multiple recent reviews [11,13].

**Figure 2.**
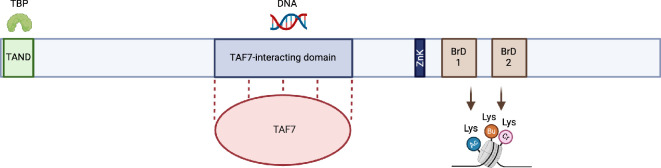
TAF1 protein domains and functions. DNA and protein interactions are shown. The TAF1 N-terminal (TAND) domain interacts with TBP to inhibit DNA binding [17]. The TAF7-interacting domain binds TAF7 to form a stable complex via a heterodimeric triple barrel (not shown); within this region is a DNA-binding domain, which forms DNA–protein interactions via a winged-helix domain [18]. Towards the C-terminal lies a Zinc knuckle domain (ZnK), which aids in the recruitment of TFIID to endogenous promoters [19]. TAF1 contains tandem bromodomains (BrD1 and BrD2) that bind acetyl-lysines and acyl-lysines, where the second bromodomain of TAF1 binds to butyryl (Bu)- and crotonyl (Cr)-lysine residues on histone tails [20,21]. The diagram shows the relative size of each protein domain, semi-to-scale. Created with BioRender.com.

TAF1 has multiple functions including forming various protein–protein interactions and has DNA-binding activities (figure 2). TAF1 functions as a scaffold, contributing to the assembly and maintaining the structural integrity of TFIID [22], and is involved in the co-translational assembly of several TAFs and TFIID [14,15,23]. TAF1 has been shown to bind the initiator element (INR) directly, which lies at the core promoter downstream (−2 to +4 bp of the transcription start site) of any TATA box present (−31 to −26 bp) [24]. TAF1 also interacts with DNA downstream of the PIC, such as at the motif ten element (MTE)/downstream promoter element (DPE) (+28 to + 32 bp) ([10,11,25]; figure 1*b*).

At the TAF1 N-terminal domain (TAND) (figure 2), TAF1 binds TBP to inhibit DNA binding in its non-promoter-bound state [17,26,27]. At its TAF7-interacting domain, TAF1 forms a stable interaction with TAF7, and a smaller winged-helix domain within this region has been shown to bind promoter DNA [18]. TAF1 also contains a conserved Zinc knuckle domain, which is involved in the recruitment of TFIID to endogenous promoters [19]. Towards the C-terminal end, TAF1 contains a unique double bromodomain, which recognizes post-translational modifications on histones and transcription factors including acetylated, butyrylated and crotonylated lysines [20,21,28]. Besides these functions, several enzymatic activities have been attributed to TAF1 in the past, which could not be confirmed in recent TFIID and/or TAF1 studies. These include kinase, histone acetylation and ubiquitination activities [29]. These studies could not be reproduced by others and much of the related work by Sauer *et al*. has been retracted [1]. Overall, TAF1 can regulate transcription through a variety of mechanisms and is probably responsive to epigenetic changes in chromatin.

### 1.2. *TAF1* messenger RNA (mRNA) variants and protein isoforms

#### 1.2.1. Alternative splicing of *TAF1*

**Figure 3.**
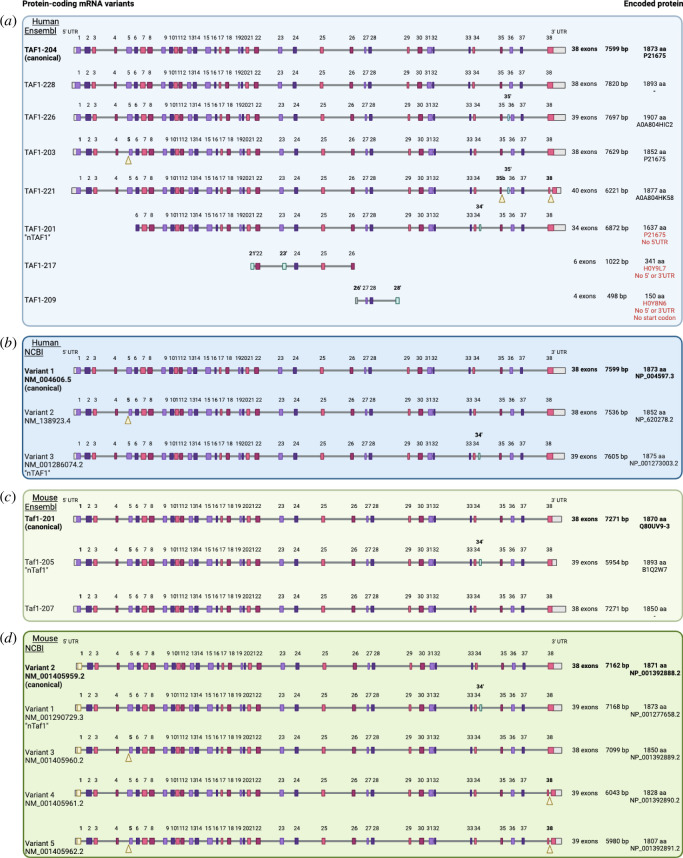
Genomic architecture (exon–intron structure) of protein-coding mRNA variants for human and mouse *TAF1*. (*a*) Eight protein-coding variants are shown for human *TAF1* as described in Ensembl and (*b*) three are defined by NCBI. (*c*) Three protein-coding variants are shown for mouse *Taf1* as categorized by Ensembl and (*d*) five are categorized by NCBI. The canonical variant (c*TAF1*/c*Taf1*; bold) and n*TAF1*/n*Taf1* are indicated. Exons are shown as coloured boxes and introns as grey lines. Grey boxes indicate 5′- and 3′-UTRs. Blue boxes indicate additional exons to the human canonical *TAF1* variant. The total number of exons, amino acid (aa) length, and their amino acid translation by UniProt ID are shown on the right. Yellow arrowheads and bold writing on exon number indicate the absence of nucleotides in exons compared with the same exons in the Ensembl canonical variant of respective human or mouse *TAF1*; yellow boxes and bold writing on exon number indicates sequence variation in exons to the respective human or mouse canonical *TAF1* variant. Red writing of UniProt ID indicates that mRNA is unlikely to be translated to protein owing to missing structural features of a complete open reading frame as indicated. Created with BioRender.com.

The human *TAF1* gene lies in Xq13.1 and produces a canonical transcript (‘c*TAF1*’) of 38 exons (Ensembl 2024, https://www.ensembl.org/Homo_sapiens/Gene/Summary?db=core;g=ENSG00000147133;r=X:71366222-71532374). c*TAF1* ^1^ is called ‘*TAF1*−204’ in the Ensembl database and is a 7599 bp transcript encoding 1873 amino acids, a 3bp stop codon, and a surprisingly short 5′-untranslated region (UTR) of 18 bp and a 3′-UTR of 1959 bp (figure 3; table 1). Ensembl describes the canonical variant as ‘the most conserved, highly expressed, has the longest coding sequence, and is represented in other key resources, such as NCBI and UniProt’ (Ensembl 2024). *TAF1*−204 is also the Matched Annotation from NCBI and EMBL-EBI (MANE) Select transcript variant, which is the ‘default transcript per human gene that is represented by biology, well-supported, expressed and highly conserved’ (Ensembl 2024).

**Table 1.** Table of likely human and mouse protein-coding transcripts as named in Ensembl, NCBI and the literature.

	Ensembl variant	NCBI variant	alternative name
**human**	*TAF1*−204	variant 1	c*TAF1*
	*TAF1*−226		
	*TAF1*−228		
	*TAF1*−203	variant 2	
	*TAF1*−221		
	*TAF1*−201	variant 3	n*TAF1*, *TAF1*−34′
**mouse**	*Taf1*−205	variant 1	n*Taf1*, *Taf1*−34′
	*Taf1*−201	variant 2	c*Taf1*
	*Taf1*−207		
		variant 3	
		variant 4	
		variant 5	

Currently, we do not know how many *TAF1* mRNA variants exist. In humans, *TAF1* has several internal exons that are found within alternatively spliced mRNA variants in various transcript databases and the literature. Ensembl describes 27 alternatively spliced transcripts (ENSG00000147133), and the NCBI database refers to 13 such transcripts (NG_012771.2 RefSeqGene) derived from multiple tissues including brain, foetal and HeLa cells. At least 10 splice variants are reported in the literature from predominantly brain samples, in which TAF1 is abundant [6,30]. In figure 3*a,b*, we show only those eight transcripts from Ensembl and the equivalent three from NCBI that are described as potentially protein-coding, i.e. in that carry an open reading frame (ORF) throughout with (mostly) complete 5′- and 3′-UTRs. Without further analysis of full-length *TAF1* transcripts in different tissues, it is impossible to know how many of these mRNAs are real. However, *TAF1*−204 (*cTAF1*), *TAF1*−228, *TAF1*−226, *TAF1*−203 and *TAF1*−221 appear bona fide because they have a full-length ORF and 5′- and 3′-UTRs. *TAF1*−201, while incomplete, potentially encodes the neuronal-specific form of TAF1 (N-TAF1 or TAF1−34′), that has been characterized at the protein level and probably corresponds to the NCBI transcript variant 3. NCBI transcript variant 1 corresponds to the canonical form of *TAF1* (*TAF1*−204) and variant 2 is *TAF1*−203. Thus, in total, six full-length probably *bona fide* protein-coding transcripts have been described in Ensembl and NCBI for human *TAF1* (table 1).

With respect to the two other *TAF1* transcripts described in Ensembl as ‘protein coding’: *TAF1*−217 (described in foetal brain and thymus) and *TAF1*−209 (origin unknown), only contain four and two canonical *TAF1* exons, respectively, plus additional alternative exons that are not described in the literature. Neither transcript is full-length and therefore it is currently impossible to know if they represent functional mRNAs. It is important for the field to determine the full complexity of *TAF1* alternative transcripts, presumably each undertaking a different function, within each cell type, and furthermore, it would be very useful to determine relative levels of *TAF1* transcripts in different tissues, conditions or at different developmental stages.

*TAF1* transcripts in online databases mostly differ in alternative exon usage, including smaller exons, primarily towards the 3′ end of the *TAF1* gene (figure 3*a*,*b*). For example, microexon 34′ (6 bp) is present in *TAF1*−201/variant 3/n*TAF1*, whereas exon 35′ (102 bp) is present in *TAF1*−221 [30,31]. Alternative exon 35b, is slightly shorter than exon 35a (126 bp versus 117 bp) because it does not contain the nine 3′ terminal base pairs [31]. Exon 35b is present in *TAF1*−221 and maintains the reading frame.

We note that some nucleotides are missing from the canonical exons in some *TAF1* variants in the databases, such as 63 bp 3′ of exon 5 in *TAF1*−203/variant 2. Similarly, *TAF1*−221 shows sequence variation within exon 38 from the canonical exon 38 and is missing 1081 bp from its 3′-UTR. It is not clear whether these sequence changes are *bona fide* or artefacts of sequencing and/or complementary DNA (cDNA) cloning, however, the changes in nucleotide numbers in the coding regions are multiple of 3 and so would maintain the ORF.

**Figure 4.**
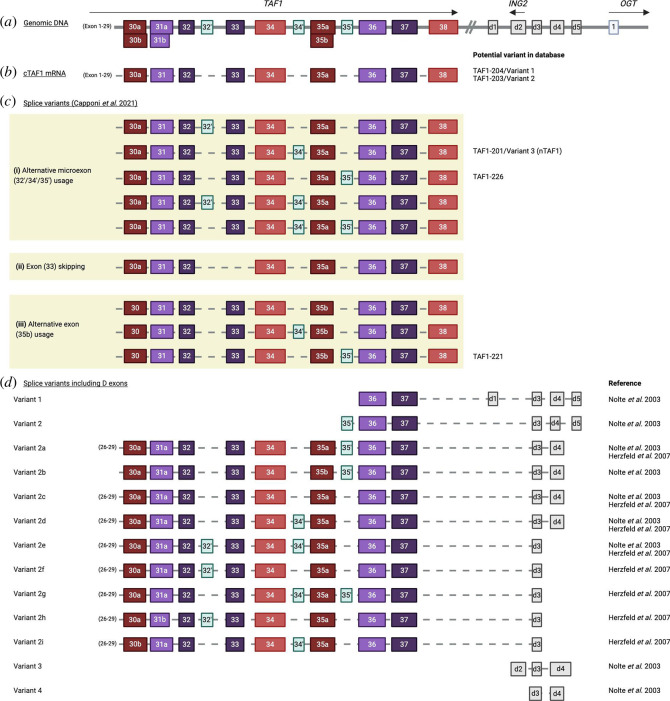
mRNA splice variants of human *TAF1* in the literature. (*a*) *TAF1* genomic DNA region (Xq13.1) including human *TAF1* exons 30–38 that have been found in mRNAs (5′ exons 1–29 are not shown). Alternative exons 32′, 34′ and 35′ and exons 35 a/b are shown in genomic DNA at their positions relative to canonical exons. *TAF1* is transcribed on the sense strand and ING2 is transcribed downstream of *TAF1* in an antisense orientation. *OGT* is transcribed in a sense orientation downstream. In older literature, d exons 1–5, shown in grey, were described as located downstream of *TAF1* [31] but whether they are spliced with canonical *TAF1* exons is yet to be robustly demonstrated. (*b,c*) mRNA variants described by Capponi *et al*. involving exon 30–38 taken from sequenced brain transcripts [30]. (*b*) Canonical *TAF1* (c*TAF1*, *TAF1*−204). (*c*) Nine further *TAF1* variants resulting from alternative splicing where (i) alternative exons 32′, 34′ and/or 35′ are incorporated; (ii) exon 33 is skipped in the c*TAF1* transcript or (iii) alternative exon 35b is incorporated into the *TAF1* transcript instead of 35a. Exons and introns are not to scale. Data were obtained from long-range reverse transcription-polymerase chain reaction (RT-PCR) in which the amplicon spanned between exons 30 and 38. Whether canonical *TAF1* exons 1−29 are present upstream of exons 30–38 cannot be confirmed, because of lack of upstream sequencing data from the transcripts other than c*TAF1*. We speculate on the presence of *TAF1*−203/variant 2 in NCBI (which is identical to c*TAF1* apart from exon 5 which is not included in the sequencing), n*TAF1* (*TAF1*−201/variant 3), *TAF1*−226 and *TAF1*−221. (*d*) mRNA variants shown are based on phage-cloned DNA and RT-PCR analyses. Variants 1−2i include *TAF1* exons, whereas variants 3 and 4 are transcribed independently of *TAF1*. Nolte *et al*. investigated transcripts using primers designed between exon 30 and d4, whereas Herzfeld *et al*. investigated transcripts using primers designed between *TAF1* exon 26 and d3. Variants identified by Nolte *et al*. and Herzfeld *et al*. were identified in the human foetal brain and caudate nucleus [31,32]. Created with BioRender.com.

Since most alternative splicing events reported in the literature occur towards the 3′-end of the *TAF1* gene, in a recent paper Capponi *et al*. amplified the region between exons 30 and 38 in cDNA derived from male and female adult human prefrontal cortex and presented the 10 most abundant transcripts ([30]; figure 4*b,c*). Although it is speculative how these published transcripts relate to the full-length transcripts described in the Ensembl and NCBI databases, one transcript appears to be derived from c*TAF1* (*TAF1*−204, variant 1) or from *TAF1*−203 (variant 2) (which are identical apart from exon 5, which is not included in the sequencing), one is potentially derived from n*TAF1* (*TAF1*−201, variant 3), one is probably from *TAF1*−226, and one is probably from *TAF1*−221 (figure 4*b,c*). By nanopore long-read sequencing in HeLa cells, Capponi *et al*. showed that approximately 70% of *TAF1* transcripts are c*TAF1*, but there is a large inter-individual variation in relative levels of expression of different variants within the human prefrontal cortex [30]. Aside from these 10 most abundant variants in the brain, they state that remaining low abundance variants are less than 1% individually and make up less than 5% on average (maximum 12%) of the remainder. Other mRNA variants include the incorporation of alternative exon 32′, which is 22 bp in length and would cause a translational frameshift leading to a stop codon in exon 34.

Older literature states that *TAF1* may function together with downstream genes such as *ING2* (antisense strand) and *OGT* (sense strand), in what has been referred to as a ‘multiple transcript system’ (MTS) [31]. This MTS has been described to also include five exons, referred to as exons d_1_–d_5_ (‘downstream’ exons 1−5), located 3′ to canonical exon 38 ([31,32]; figure 4*a*). Exons d_1_–d_5_ were reported as spliced with *TAF1* exons (26−37 but not exon 38) to produce at least eight variants in the human brain (‘variants 2a-i’) (figure 4*d*). However, while 13 variants containing d exons are presented in the NCBI database, they are described as non-coding RNA [31–33].

The prevalence and function of possible ‘d’ mRNA variants are not known [30]. While the 38 canonical exons are highly conserved across vertebrates and invertebrates, the five d exons are less conserved and—if real—predominantly occur in primates [32]. Expression of d_2_–d_3_ and d_3_–d_4_ was reported to be higher in the brain than in other tissues, where disease-related sequence changes in d exon expression were reported to disturb dopamine transport and metabolism [33]. However, recently, Aneichyk *et al*. could not confirm the presence of these transcripts and did not find MTS exons to be transcribed with *TAF1* exons using *de novo* transcript assembly in fibroblasts and neural cells [4]. Together, it is unlikely that d exons produce functional mRNAs.

#### 1.2.2. Human TAF1 protein isoforms

While many *TAF1* transcript variants exist in the databases and the literature, not all may be *bona fide* mRNAs that are translated into a functional protein. For example, some variants may include retained introns from partially processed mRNAs, potentially resulting in nonsense-mediated decay (NMD) [34,35]. However, as shown in figure 3*a,b*:

- five Ensembl ‘protein-coding’ mRNA variants (*TAF1*−204 [c*TAF1*], -228,-203, - 206 and -221) have intact 5′- and 3′-UTRs, start and stop codons, and a full-length ORF; and
- the three protein-coding transcript variants in NCBI share homology with three Ensembl variants (*TAF1*−204, variant 1; *TAF1*−203, variant 2; *TAF1*−201, variant 3) (table 1). The sequence homology of the three full-length Ensembl/NCBI transcripts supports their legitimacy.

However, differences in total transcript lengths are shown, such as for *TAF1*−203/variant 2, due to variable 5′- and 3′-UTR lengths probably owing to different sources providing the sequencing data. Furthermore, protein lengths in online databases vary: for example, the canonical form of human TAF1 is 1893 amino acids in UniProt (P21675) but 1873 amino acids would be translated from the Ensembl *TAF1*−204 (canonical) mRNA transcript. The updated sequence on NCBI is 1873 amino acids (NP_004597.3), corrected from the previous version (NP_004597.2). The additional 20 amino acids in UniProt were previously reported, but it now appears that this may have been caused by an error in the prediction software of UniProt. The extra 20 amino acids would result from an ATG, that lies in the 5′-UTR and is upstream from cognate ATG start. Furthermore, the second ATG in the genetic sequence (but not the first upstream) lies in the consensus Kozak sequence, which would direct translation initiation to the second methionine. Therefore, the current evidence suggests that the correct length of human cTAF1 is 1873 amino acids.

Transcripts containing alternative exon 32′ have been published [30]. However, exon 32′ is not in any of the protein-coding transcripts in published databases but is present in non-coding transcripts such as *TAF1*−218 (NMD, ENST00000485087.6), *TAF1*−211 (retained intron, ENST00000468167.6), *TAF1*−223 (NMD, ENST00000683358.1) and *TAF1*−220 (NMD, ENST00000682124.1). We note that exon 32′ is 22 bp in length and would produce a frameshift and early stop codon resulting in a truncated protein. Finally, transcripts containing d exons do not fulfil the criteria for encoded proteins, since many have incomplete 5′-UTRs and are probably degraded by NMD (ENSG00000147133).

#### 1.2.3. Neuron-specific TAF1

The relative abundance of each of the various transcripts in different tissues and their function have not been comprehensively described and, importantly, other splice isoforms may yet be found in different tissues. However, one transcript, n*TAF1*/*TAF1*−201 (Ensembl)/variant 3 (NCBI), is of particular interest because it appears to be neuron-specific [6]. *TAF1*−201 fulfils the criteria for ‘protein-coding’ but has no 5′-sequence before exon 6, which is presumably an artefact, whereas the homologous NCBI variant 3 transcript has an identical coding sequence; both mRNAs include the 6 bp microexon 34′, which encodes an alanine and a lysine residue (figure 3*a,b*). While direct protein expression data on this neuron-specific isoform are scarce, in part because no specific antibody is currently available, homology has been found in the mouse in which transcript *Taf1*−205/variant 1 incorporates the same microexon (figure 3*c,d*), indicating that both species produce functional N-TAF1 protein. Note that, confusingly, the mouse and human transcript numbers do not correspond, i.e. human *TAF1*−201 is not the homologous transcript to mouse *Taf1*−201.

An antibody made with an epitope across canonical exons 34–35 (towards the C-terminal), detected (by Western blot) the N-TAF1 isoform in human 293 T cells that were transiently transfected with green fluorescent protein (GFP)-TAF1−34ʹ cDNAs [30,36]. A mouse protein containing this epitope was detected in the cortex and striatum of the mouse brain by immunohistochemical staining using the same TAF1−34ʹ-specific antibody [36]. In addition, long-range reverse transcription-polymerase chain reaction (RT-PCR) showed that the identical 6 bp microexon 34ʹ sequence is incorporated into mouse *Taf1* transcripts at this conserved region [37]. Long-range PCR methods used to generate these data involved first-strand synthesis from RNAs by long RT and subsequently fragment PCR by use of the long RT products (i.e. cDNA) as a template [6,37]. Thus, whether other microexon 34ʹ containing transcripts are amplified by these methods is uncertain. However, since discrimination of microexon-containing mRNAs from canonical mRNAs is challenging owing to the small size of such microexons, Capponi *et al*. used *in situ* hybridization to discriminate between c*TAF1* and n*TAF1* using the BaseScope method with specific probes designed against the 6 bp microexon 34ʹ or the sequence spanning the flanking exons [36]. This revealed the presence of the microexon experimentally, but this RNA-based analysis did not analyse the sequences 5ʹ and 3ʹ of the microexon-containing region and thus does not demonstrate the transcript length.

Capponi *et al*. provided evidence for microexon 34ʹ incorporation into the n*TAF1* transcript by neuron-specific splicing factor Serine/Arginine Repetitive Matrix 4 (SRRM4) [30,36] and Cirnaru *et al*. reported that in rat striatum, micro RNA (miRNA) knockdown (KD) of n*Taf1* by intracerebroventricular adeno-associated virus (AAV) injection successfully reduced N-TAF1 expression as shown by Western blot of tagged GFP [38]. It remains unclear whether microexon 34ʹ is incorporated into other transcripts/protein isoforms.

#### 1.2.4. Mouse *Taf1* splice variants and protein isoforms

cTAF1 protein sequences are highly conserved between mouse and human, with an amino acid sequence identity of approximately 96% (UniProt: Q80UV9 versus P21675, Release 2024_01). Mouse genes tend to have fewer transcripts than their human orthologues; in Ensembl, mouse *Taf1* has seven transcripts, and NCBI indicates that there are five *Taf1* transcripts (figure 3*c,d*). Of the Ensembl transcripts, three are probably full-length *bona fide* transcripts, having 5ʹ- and 3ʹ-UTRs and a long ORF and appear to correspond to human c*TAF1* (mouse *Taf1*−201) and n*TAF1* (mouse *TAF1*−205) (table 1; figure 3*a,c*). In Ensembl, compared with the human canonical transcript (*TAF1*−201), mouse transcripts show sequence variation at exon 1 and exon 5 (*Taf1*−201 and −207). Meanwhile in NCBI, mouse transcript variants 3 and 5 contained the shorter form of exon 5 present in human *TAF1*−203/variant 2, and mouse variants 4 and 5 contained the sequence variation in exon 38 that is present in human *TAF*1−221 (figure 3*d*). Conservation of these sequences between mice and humans supports the legitimacy of these findings.

## 2. TAF1 and development and disease

### 2.1. TAF1 function and experimental loss of function

A large body of data suggests that TAF1 is essential for embryonic development [3,39,40]. Moreover, TAF1 and other TFIID components may play key roles in cell proliferation and growth, such as TAF1 in the G1 phase of the cell cycle [41,42]. Effects may be cell state specific, e.g. expression of TAF1 and other TFIID components (TAF4 and TBP) is higher in myoblasts (muscle cell progenitors) compared with myotubes (differentiated muscle cells) [43]. Stemness seems to be regulated by TFIID TAFs [44]. In addition, TAF1 has been reported to bind to PAX3 (regulator of myogenesis), leading to its proteasomal degradation [45]. This is similar to another canonical TFIID complex component, TAF4, for which expression in rat embryonic cortical neural stem cells (NSCs) was strong and decreased with neuronal (but interestingly not glial cell) differentiation [46].

In mice, TAF4 is required for mid-stage gestation and the expression of paralogues TAF4A and TAF4B overlaps at early embryonic stages [47]. TAF4B is also highly expressed in embryonic stem cells (ESCs) and is downregulated during differentiation, but in contrast to TAF4, maintains rather than inhibits ESC proliferation and cell cycle progression [48]. Knockout (KO) of *Taf4a* in mouse ESCs prevented the completion of differentiation into glutamatergic neurons and cardiomyocytes owing to impaired PIC formation at the promoters of critical differentiation genes [47]. These findings highlight the roles of TFIID components in regulating stemness and cell differentiation—key processes in embryonic development.

Homozygous KO of *taf1* in zebrafish is embryonically lethal and affects neurological, musculoskeletal and cardiac development. Null embryos have upregulation of cardiac and muscle cell differentiation as shown by RNA-seq analysis [40]. To understand the role of TAF1 in development including in the brain, *taf1* gene expression was knocked down in zebrafish by injection of the embryo at three days post fertilization with a morpholino to *taf1* [3]. Similar to full KO, *taf1* KD was embryonically lethal, and both full KO and KD fish had neurodevelopmental defects and reduced area of the optic tectum (midbrain) indicating microencephaly [3,40]. In the *taf1* KD zebrafish, overexpression of wild-type(WT) human *TAF1* mRNA restored the area of the optic tectum to WT levels, and overexpression alone did not induce a phenotype that was significantly different from that of controls [3]. Furthermore, in neonatal mouse ventricular cardiomyocytes (immature cardiac tissue), chemical TAF1 bromodomain inhibition led to transcriptional changes in genes regulating mitochondrial function, cell proliferation, cell senescence, apoptosis, cell polarity, cell differentiation and congenital malformations [49]. These findings indicate that TAF1 is essential to embryo viability and has an important function in growth that may involve the regulation of stemness.

Gene editing of *Taf1* at postnatal day three in rats using CRISPR/Cas9 technology produced behavioural and motor defects at neonatal and juvenile periods of development (days 21, 23 and 35 after birth) with histopathological effects in the cerebellum and cerebral cortex [50,51]. The abnormal neuronal morphology and Purkinje cell apoptosis were found to be owing to decreased CaV3.1 T-type channel expression, and neuropathology and behavioural defects in *Taf1*-edited rats were rescued by induction of the T-type calcium channel enhancer SAK3 (injected at postnatal day 21) [50,52]. Similarly, early-stage AAV–miRNA-induced *Taf1* KD in mice and in rats produced motor deficits, with a stronger phenotype from *Taf1* KD at postnatal stage P0 compared with at three weeks after birth [38], highlighting a role of TAF1 in early development. These data indicate that complete loss of function *TAF1* mutations is embryonically lethal, whereas less detrimental missense or KD mutations may allow survival with neurodevelopmental and cardiac defects.

It is possible that different TAF1 protein isoforms play specific roles at different stages of development. For example, in mice, n*TAF1* has higher expression postpartum than in early embryonic development, whereas *Taf1* mRNA variants without microexon 34′ are most highly expressed during early development [37]. Moreover, n*TAF1* showed higher relative expression in brain tissue compared with neuroblastoma [37] and microexon 34′ incorporation into mRNAs is reported to be highest in post-mitotic neurons [36], suggesting that n*TAF1* plays a greater role in differentiated neurons than in proliferating cells.

Finally, we have recently submitted a report on the generation and characterization of the first conditional *Taf1* loss of function model in mice [53]. Similar to zebrafish KO/KD models, *Taf1* KO was embryonically lethal, producing no hemizygous males or homozygous females. Furthermore, *Taf1* was essential for development at an early embryonic stage, as blastocysts with complete *Taf1* KO were able to survive to stage E3.5 but not E9.5. Intriguingly, female (carrier) mice heterozygous for the *Taf1* KO allele were produced with equal *Taf1* expression to WT female mice but showed behavioural deficits and increased body weight as adults (12 months old). These findings indicate that heterozygous *Taf1* KO may have skewed X-inactivation and that possibly having one functional copy of the gene may affect brain function, even though gene expression appears to be maintained overall; perhaps this is owing to a subset of neurons or cell types affected by *Taf1* deficiency.

### 2.2. Human *TAF1* mutations and development

In humans, males with congenital hemizygous mutations in *TAF1* (maternally inherited or *de novo*) show cardinal facial dysmorphologies including microencephaly, with intellectual disability from a young age [3,40]. Comparable microencephaly and intellectual disability have been reported for hypomorphic mutations in other canonical TFIID components such as TAF2 (8q24.12) [54] and *TAF13* (1p13.3) [55], which are both inherited in an autosomal-recessive manner. Furthermore, missense variants in *TAF1* cause congenital heart disease [56], indicating that TAF1 function is required for cardiac development.

**Figure 5.**
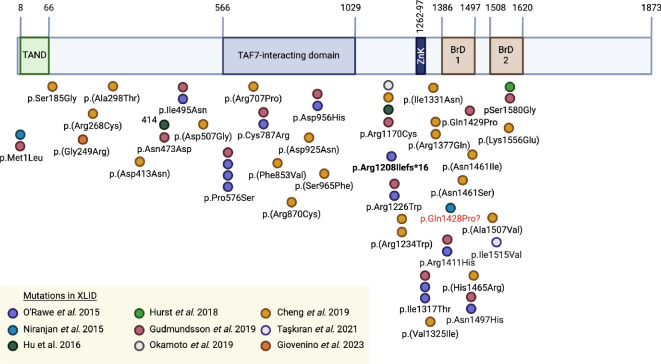
TAF1 protein-coding variants in human XLID. Amino acids (aa) in TAF1 are numbered marking the start and end of each major protein domain. Congenital mutations in *TAF1* identified in XLID are shown, where aa locations in relation to the protein domains are indicated; colour indicates the source reference. Of note, mutations related to other phenotypes such as congenital heart disease are not shown. Protein domains are scaled to align with corresponding exons, where aa numbers are defined by the most updated accession reference for the human TAF1 protein on NCBI (NP_004597.3). The only reported frameshift mutation is shown in bold text. One reported mutation does not match with the aa in the protein sequence and is shown in red text (p.Gln1428Pro). Of note, only male patients are shown for consistency with phenotypic effects of XLID. TAND, TAF1 N-terminal domain; TAF7, TATA-Box binding protein associated factor 7; Zn, Zinc knuckle domain; BrD, bromodomain. Created with BioRender.com.

The severity of neurodevelopmental effects from mutations in *TAF1* could depend on the location of the mutation in the gene, and thus the protein function affected, but missense mutations occur throughout the protein sequence in people with XLID (or suspected XLID) and no single common mutation or domain is affected. However, fewer mutations are reported at the C-terminal than at the central portions of the protein, where none have been reported to occur after the second bromodomain (BrD) (figure 5; [3,40,57–62]). The reason that no affected individuals with C-terminal mutations have been described may be because missense mutations towards the end of the gene might not affect overall protein function and would not result in a clinical phenotype. In line with this, the region C-terminal to the BrDs lacks both structure and evolutionary conservation [2]. Alternatively, this portion of TAF1 may be essential to its function and the viability of embryos, and thus few living patients with such mutations have been reported owing to early lethality. Further mutational analyses are required to demonstrate the effect of *TAF1* mutations on each protein domain in humans.

The predicted regions of the TAF1 protein affected by congenital *TAF1* mutations in XLID patients are shown in figure 5. For example, O’Rawe *et al*. describe eight missense *TAF1* variants leading to XLID, four of which change conserved residues reported as regions important for TAF7 binding [3]. Three separate substitutions (p.Cys787Arg, p.Pro576Ser or p.Asp956His) in different families occurred within the central TAF7-interacting domain (amino acids 566−1029). For example, the p.Cys787Arg substitution, where the polar cysteine residue is replaced with the large basic arginine, is predicted to destabilize the heterotrimeric triple-barrel-fold comprising TAF1 and TAF7, altering the interaction between these two proteins. Similarly, p.Asp956His leads to an acidic-to-basic residue change, disrupting the glycine-rich motif and probably altering TAF7 binding. Interestingly, Cheng *et al*. report the difference in Gibbs free energy change for XLID-associated mutations in the TAF1-TAF7 binding domain compared with the WT molecule, where many mutations such as p.Gly680Asp (reported in one female only), p.Phe853Val and p.Arg870Cys showed no differences, whereas p.Arg707Pro shows a positive change indicating that it destabilizes the protein structure [58]. Gudmundsson *et al*. reported that the pArg1170Cys (c3568C > T) variant is probably disease-causing and deleterious owing to its effects on a conserved base, which has been reported in four separate XLID studies [40,58,60,61].

Cheng *et al*. described variants in the TAF1 double BrD, and a large proportion of mutations have been located here by multiple XLID studies [3,40,57,58,60]. However, generally missense mutations in this region do not appear to change the structural stability of TAF1, and, therefore, whether these mutations produce *TAF1* loss of function effects and how they impact development and produce clinical phenotypes is difficult to predict [58]. Details of other XLID-associated *TAF1* mutations and their predicted effects on protein structure and function are described in O’Rawe *et al*. [3] and Cheng *et al*. [58].

Finally, upon screening patients with missense mutations in *TAF1* who have congenital heart disease and neurodevelopmental defects, the most deleterious missense variants usually occurred within the functional domains of TAF1, including the N-terminal domain, TAF7-interacting domain, DNA-binding domain and BrD (in 12 of 16 cases) [49]. Interestingly, similarly to XLID patients, no *TAF1* mutations related to congenital heart disease were reported in the gene after exon 31 (p.Lys1576Glu), indicating that mutations at the C-terminal end were not found in patients with this phenotype [56]. In such instances where TAF1 protein function and protein–protein interactions are altered, gene expression during development is likely to be perturbed to produce XLID and cardiac phenotypes.

## 3. TAF1 in neurodegeneration

Defects in RNA pol II-mediated transcription can lead to multiple types of motor disorders and other features and these diseases have been called ‘transcription syndromes’ [63]. For example, spinocerebellar ataxia 17 is an inherited progressive movement disorder primarily affecting the cerebellum, which is caused by an expanded polyglutamine tract in TBP [64], altering its interaction with TFIID and dysregulating expression of its target genes [65,66]. Mutations causative for one form of familial amyotrophic lateral sclerosis (ALS)—an adult-onset neurodegenerative disorder leading to loss of upper and lower motor neurons—lie within the RNA/DNA-binding protein fused in sarcoma/translocated in liposarcoma (FUS/TLS) [67,68], which can interact with TBP and may influence RNA polymerase II and the TFIID complex assembly, among other roles in transcription [69,70]. Of note, TBP is also known to regulate pol I- and III-mediated transcription [28]. Thus, we cannot say for certain that these motor disorders are directly caused by TFIID defects. Many defects in TFIID and other components of the basal transcription machinery result in neurodegeneration, but these are beyond the scope of this review as we focus here on TAF1.

Neurodegenerative diseases implicating TAF1 dysfunction in their pathogenesis predominantly exhibit atrophy of the striatum or other regions of the basal ganglia with motor defects. These include, but may not be limited to, XDP, HD and Parkinson’s disease (PD).

### 3.1. X-linked dystonia–parkinsonism

XDP is an adult-onset neurodegenerative disorder affecting individuals with ancestry from the Philippines. XDP predominantly affects males owing to its X-linked mode of inheritance and it arises from a presumed founder mutation with a prevalence of 0.57 per 1 00 000 on the island of Panay, Philippines [71]. In most patients, XDP is characterized initially by dystonia (93% of cases) that generalizes within 5–10 years from the age of onset in males, which eventually transitions into parkinsonism with a mean age of death of 55.6 years (range: 33–81) [71,72]. Relatively few XDP female cases (approx. 14 symptomatic to date) have been reported with similar XDP features to affected males but with a later age of onset (males: approx. 39 years (range: 12–64 years) versus females: approx. 52 years (range: 26–75 years)) [8,71,73,74]. Similar to other major movement disorders, the basal ganglia are affected in XDP. In the striatum, medium spiny neurons are preferentially lost from the caudate nucleus and putamen [7,75] in a similar manner to that shown in HD [76] and cerebral ischaemia [77]. Additionally, it has been reported that neural progenitor cells are lost from the subventricular zone [75]. Thus, although XDP is a rare inherited form of dystonia and parkinsonism, it may share a similar neuropathological mechanism to other late-onset neurodegenerative conditions.

In 2003, Nolte *et al*. first identified a 300 kb interval of Xq13.1 associated with XDP, described as the MTS [31]. This region encoded eight genes, including *TAF1*, and sequencing within the affected haplotype showed five single-nucleotide changes and a 48 bp deletion [31]. Further analysis identified an approximately 2.6 kb SINE-VNTR-Alu (SVA) type F retrotransposon insertion in intron 32 of *TAF1* [6]. Of these seven sequence variants, three fall within *TAF1* introns, while the remaining four are localized to an intergenic region containing the MTS 3′ to *TAF1*. Domingo *et al*. narrowed the disease locus in the XDP haplotype to a 294 kb region that included four genes: *TAF1*, *OGT*, *ACRC* and *CXCR3* [78]. Subsequently, Aneichyk *et al*. revealed 47 novel variants that segregated with XDP, which were confined to a narrower minimal critical region of 203.6 kb, encompassing only *TAF1* [4].

The SVA insertion in intron 32 of *TAF1* appears to be causative for XDP. This is primarily shown by the inverse correlation between the number of hexameric repeats ((CCCTCT)_*n*_, *n* = 30–55 repeats) within the SVA and both the age of disease onset [5,79] and with *TAF1* expression [79]. Repeat expansion in somatic tissues has been shown to be repeat length-dependent and may be tissue-specific, with greater levels of expansion in the brain than in blood [80]. Differences in hexameric repeat numbers between brain regions may also play a role in selective neurodegeneration [81].

**Figure 6.**
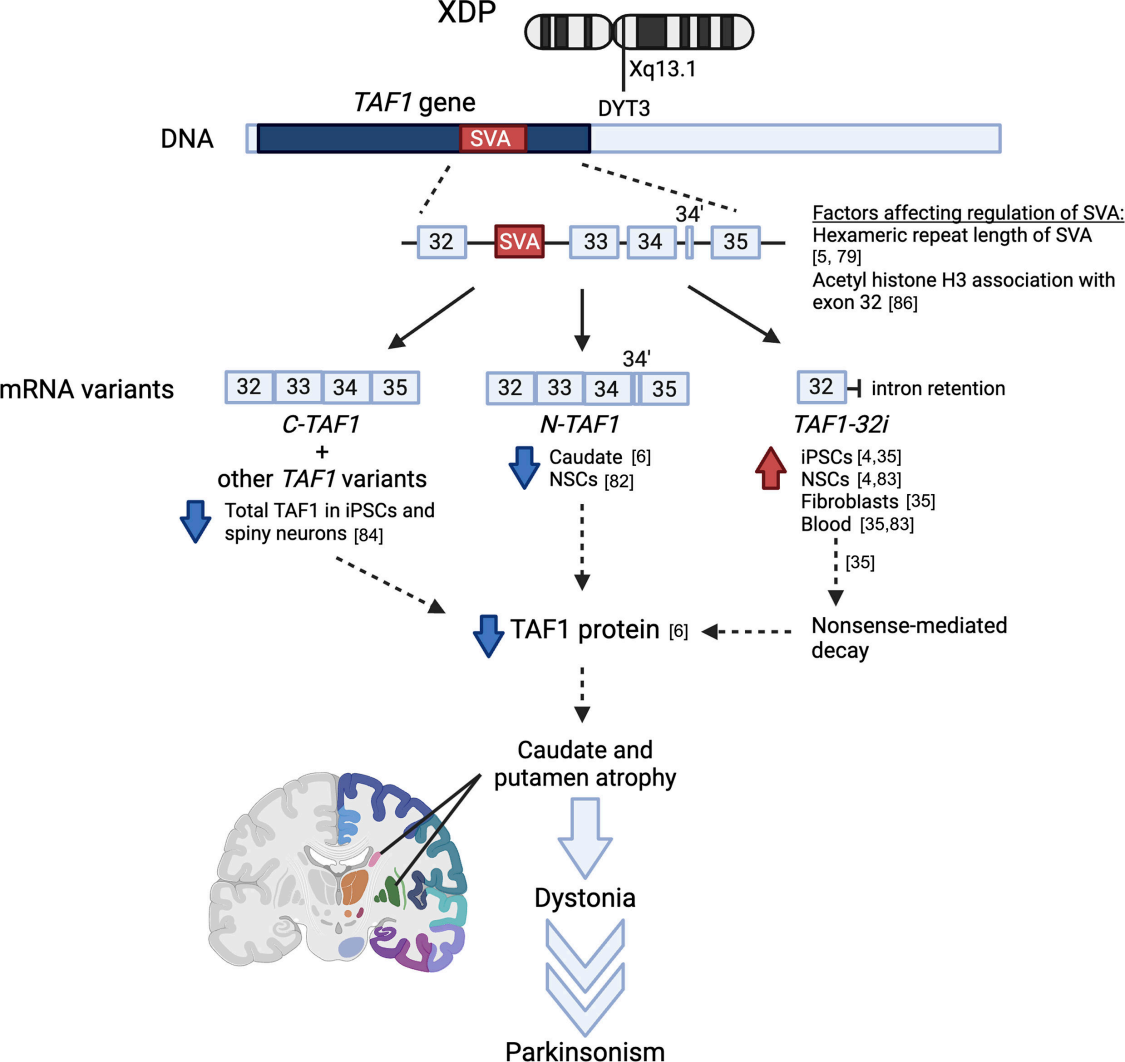
Evidence linking *TAF1* to XDP pathogenesis. The region of Xq13.1 affected in the XDP allele is at the MTS, in which *TAF1* lies. *TAF1* from XDP patients or XDP carriers contains a novel SVA within intron 32, and *cTAF1*, *nTAF1* and *TAF1−32i* show differential transcript levels in XDP brain compared with that without XDP. Changes to transcript levels may affect TAF1 protein levels in yet unknown ways (dotted arrow), including NMD of the transcript, preventing synthesis of TAF1 proteins. Other mechanisms of differential *TAF1* expression may be owing to the length of the hexameric repeat in the SVA and the association of acetyl histone H3 with exon 32. Nonetheless, eventually the caudate nucleus and putamen regions of the neostriatum show atrophy, which is thought to be linked to the onset of dystonia and parkinsonism observed in XDP patients. Figure (introns and exons) not drawn to scale. Created with BioRender.com.

The SVA insertion in *TAF1* in XDP leads to aberrant splicing of *TAF1* isoforms ([5,6]; figure 6). Early analysis of an XDP brain showed reduced transcript levels of n*TAF1* as detected by Taqman probes [6,82]. However, a recent study indicated that the presence of the SVA may not be causal for reduced microexon 34′ incorporation into *TAF1* transcripts in XDP, since levels of microexon 34′-containing transcripts were no different between control and XDP brains [30]. Nonetheless, mice injected postnatally (P0) with miRNA knocking down *nTaf1* expression showed motor defects at two and four months of age compared with age-matched controls, with similar but fewer effects when the same experiments were performed in rats. No increase in deficits was seen in a model knocking down both *cTaf1* and *nTaf1*, indicating that *cTaf1* plays a smaller role in this phenotype, with milder effects in rats injected at later stages (three weeks old), alongside cholinergic striatal dysregulation [38].

Conversely, a novel transcript named *TAF1−32i* identified by Aneichyk *et al*. is elevated in XDP owing to the activation of a cryptic exon (32i) in intron 32, which lies just 5′ of the SVA insertion [4,83]. Misincorporation of this intronic exon causes disruption of the ORF and premature translation termination leading to NMD of the transcript [35]. Finally, the SVA has been proposed to physically impede RNA pol II elongation by forming G-quadruplexes [5], which may interfere with transcriptional elongation or cause premature termination that could affect the expression of *TAF1* variants and splicing particularly of intron 32 [4]. However, owing to the lack of XDP patient samples and ethnically matched control subjects, other pathological transcript variants of *TAF1* may have not been identified yet and they could contribute to XDP.

Although XDP is not thought to be owing to a simple loss of function of *TAF1*, reduced total *TAF1* at the mRNA [84,85] and protein [6] levels has also been reported in XDP. However, this is not consistent across studies, tissues, brain regions and cell types, and possibly changes with disease duration [4,84,85]. Nonetheless, excision of the SVA has been shown to rescue *TAF1* expression, intron retention and aberrant splicing [4,84] in human induced pluripotent stem cells (iPSCs) and NSCs. These studies indicate the XDP-SVA modifies the expression of *TAF1* variants, although owing to small patient numbers and the limitations with *post-mortem* samples, it has been a challenge to link variant expression with disease onset in a temporal manner.

In addition to gene expression changes, the effects of the SVA could be related to the chromatin state of the *TAF1* gene. For example, acetylation of histone H3 (AcH3) was reported to be modified in XDP-derived fibroblasts, such as increased AcH3 association with *TAF1* exon 32 which is upstream to the SVA, as well as downstream exon 38, and increased AcH3 association was reported at exon 17 [86]. Nonetheless, AcH3 association at exon 32 could be normalized by CRISPR/Cas9-mediated excision of the SVA [86]. However, while *TAF1* loss of function may partially explain the XDP phenotype, how dysregulation of the chromatin state of the *TAF1* locus would cause XDP pathogenesis remains to be elucidated. Interestingly, alterations in histone acetyltransferase and histone deacetylase levels and activity have been reported in progressive neurodegenerative conditions in human patients and animal models of HD [87], Alzheimer’s disease [88], PD [89], ALS [90] and spinal muscular atrophy [91].

### 3.2. Huntington’s disease

Similar to XDP, HD is a monogenic basal ganglia disorder with the first signs of pathology in the striatum [92]. Motor symptoms of HD include chorea as well as dystonia; chorea is rarely present in XDP [71]. HD is caused by a polyglutamine (polyQ) tract expansion of a CAG repeat of the huntingtin (*HTT*) gene [93]. Expansion of the HD CAG repeat is negatively correlated with age of disease onset and there is strong genetic evidence to suggest that the somatic expansion of the *HTT* CAG repeat specifically drives the timing of HD onset [94,95]. There is some overlap in genes (e.g. *MSH3*, *PMS2*) that have been implicated as genetic modifiers of both HD [95] and XDP [96]. These modifiers are implicated in repeat instability indicating a possible mechanistic link between XDP and HD. The interaction of mutant huntingtin with SP1 and TAF4 reduces the expression of dopamine receptor D2 (*DRD2*) [97], a gene that may also be downregulated in XDP [6]. Interestingly, a recent paper has shown that TAF1 expression is downregulated in the striatum of HD brains [9]. These findings indicate that both repeat expansion and dysregulation of TAF1 may have common functional features with HD, which connect these two neostriatal neurodegenerative conditions. Additionally, TAF7, which is a direct interactor of TAF1 within TFIID, is differentially expressed in HD patients [98]. However, whether TAF1 function is dysregulated in HD has not yet been determined. Additionally, while HD pathology is caused by protein aggregation of mutant HTT [99], whether aggregation of protein isoforms of TAF1 or other proteins in XDP occurs is not known.

### 3.3. Parkinson’s disease

PD is a movement disorder, in which degeneration of nigral dopaminergic neurons occurs, leading to widespread neurodegeneration and diffuse Lewy body deposition [100,101]. Unlike XDP and HD, PD is not usually an inherited disorder, although genetic forms of the disease exist [102]. Degeneration of substantia nigra, which is well known to occur in PD, has not been reported at the Parkinsonian stage of XDP [7]. However, XDP, HD and PD are neurodegenerative conditions sharing a similar disease manifestation and heterogeneous age of onset most frequently in middle age. A recent study identified a rare frameshift mutation (c.29_53dupGGA(CAG)_2_CTACCATCA(CTG)_2_C; p.A19Dfs^∗^50) in *TAF1* in two male patients from unrelated families with PD in a Chinese population [103]. Whether similar mutations in *TAF1* exist or splice variant abundance changes in other populations with PD has not been apparent so far. Currently, there is limited evidence for differential regulation of *TAF1* in PD.

## 4. TAF1 in other diseases

Diseases of TAF1 dysfunction are largely but not exclusively limited to the brain and heart and, therefore, *TAF1* expression is mostly investigated in these tissues. However, *TAF1* expression is also clearly required for early growth and development, potentially within different stem cells. Thus, TAF1 may be differentially expressed in clinical conditions such as cancer and there may be cell type-specific roles of TAF1 and its isoforms.

### 4.1. Cancer

Multiple TAFs have been shown to be mutated or dysregulated in cancer, including TAF1, TAF2, TAF4/TAF4B, TAF6, TAF9, TAF10 and TAF12 [104]. TAF1 plays key roles in processes that are tumorigenic when disrupted, such as regulation of the cell cycle. For example, it has been reported that TAF1 phosphorylates P53 (at Thr55) *in vivo* to induce G1 progression [41]. TAF1 was also linked to phosphorylation of the protooncogene MDM2 [105], leading to the degradation of P53 [41]. Downregulation of TAF1 activity leads to P53 phosphorylation (at Ser15) and activation of a DNA damage response and cell cycle arrest [106]. Thus, the transcriptional machinery has been linked with cell cycle regulation through the DNA damage response, in which TAF1 is thought to play a key role in the progression of the G1 phase of the cell cycle.

A characteristic of cancer cells is an evasion of apoptosis and TAF1 has been linked to the regulation of oxidative and genotoxic stress-induced apoptosis through control of p27^Kip1^ expression [107] indicating that TAF1 inhibition may be oncogenic in some cases. Overall, the majority of studies, albeit limited, indicate that TAF1 has a pro-tumorigenic rather than a tumour-suppressive function in cancer.

Findings from clinical data are consistent with the role(s) of TAF1 in tumorigenesis. For example, TFIID has been shown to be overexpressed in human lung and breast carcinomas [108]. Frameshift mutations have been found in *TAF1* in colorectal cancer (3.8%) with high microsatellite instability in the Korean population [109]. Mutations acquired following neoadjuvant chemotherapy are known as ‘cancer driver genes’, which are absent from paired pre-treatment samples. In oesophageal adenocarcinoma, these included a mutation in *TAF1* (c.1237G > C/p.Asp413His) [110]. TAF1 is implicated in prostate cancer pathogenesis owing to its regulation of the androgen receptor (AR), which represents a sterol receptor that requires multiple components of the transcription machinery to regulate its target genes in the prostate. TAF1 was found to increase with the duration of androgen withdrawal in patient samples indicating that it plays a role in castration-resistant prostate cancer [111]. TAF1 binds to the AR resulting in enhancement of AR transcriptional activity [111]. In uterine serous carcinoma, seven *TAF1* mutations were identified in unmatched tumours, most of which lay in the TAF7-interacting region, but the functional outcome of these mutations was not investigated [112]. Thus, TAF1 is likely to play roles in a variety of cancers although mechanisms are yet to be elucidated. Furthermore, TAF1 activity may affect tumorigenesis in a cell type- and tissue-specific manner, which is yet to be defined.

## 5. Knowns and unknowns with TAF1 activity

While many of the genetic and protein functional consequences of TAF1 expression and dysfunction have been reported as described above, there is still much to uncover regarding the relationship between TAF1 and disease. Since *TAF1* is located on the X chromosome, X-linked diseases in males have been described, whereas there are fewer studies on carrier females of *TAF1* mutations and XDP. Is TAF1 function compensated in heterozygous females? Can the testis-specific paralogue of *TAF1*, *TAF1L* (encoding TAF1L; TBP-associated factor 1 like; TAF(II)210), substitute for its function in some cases despite its restricted expression pattern under normal circumstances? Moreover, *TAF1* expression in different cell and tissue types has not been thoroughly investigated. Current knowledge and implications of TAF1 regulation in different conditions are discussed in the following sections.

### 5.1. X-inactivation in carrier females of TAF1 diseases

In both XLID and XDP, the primary affected individuals are hemizygous males, owing to the X-linked recessive nature of these rare conditions. The majority of the few female cases of XDP reported were heterozygous for the XDP allele and only one homozygous female has been reported to date ([74]; table 2). Most XDP carrier females do not have severe symptoms, possibly owing to skewed X-inactivation of the mutated allele, and many mild cases were only identified owing to genetic testing of female relatives of affected males. However, some heterozygous XDP females are highly affected with predominantly parkinsonism symptoms. Furthermore, it has been reported that X-inactivation can be skewed in favour of the XDP allele in some cases [74], where heterozygous females can show a similar phenotype to that of hemizygous males [114]. However, many more unreported cases of XDP may exist in females with mild symptoms and it has even been proposed that XDP may contribute to the apparently relatively higher proportion of PD in females in the Philippines (1 : 1.07 males : females) compared with globally (1 : 0.52 males : females) [114,118]. However, one cannot rule out ethnicity-specific effects at this moment [118].

**Table 2.** Females with mutations in the XDP disease-specific region or the *TAF1* gene and their associated phenotypes. (For XDP, patients are listed in order of age of onset, whereas patients with congenital *TAF1* mutations are listed in order of genetic changes (location of mutation along the amino acid sequence). For XDP, most cases are heterozygous (or presumed if not stated), whereas there is one case with homozygous XDP alleles as stated. For the cases with congenital *TAF1* mutations, most mutations are missense mutations (described), whereas there is one case of frameshift mutation as stated.)

predicted genotype	genetic changes/X-inactivation status	clinical presentation	age of onset (years)	affected family members	reference
heterozygous XDP	3 XDP-specific mutant alleles at DXS8030, DXS8101, and DXS559	mild chorea	26 y	mother, maternal uncle, aunt, great-grandfather	[74]
heterozygous XDP	3 XDP-specific mutant alleles at DXS8030, DXS8101, and DXS559	parkinsonism: upper limb action and postural tremor, mild breakdown of limb rapid alternating movements (RAMs), mild retropulsion	35 y	1 brother	[74]
XDP	no data	leg cramps, face dystonia, generalized in 2 years	37 y	1 father, 2 brothers	[71]
heterozygous XDP	3 XDP-specific mutant alleles at DXS8030, DXS8101, and DXS559	perioral tremor, mild impairment of tandem gait	42 y	mother, 3 brothers	[74]
heterozygous XDP	3 XDP-specific mutant alleles at DXS8030, DXS8101, and DXS559	parkinsonism: breakdown of limb RAMs, shuffling gait, cervical dystonia	42 y	mother, 4 brothers	[74]
XDP	no data	leg dystonia, generalized within 4 years	47 y	1 sister	[71]
XDP	no data	slurred speech, gait problems, then dystonia with parkinsonism	48 y	uncles on both sides, brothers	[71]
XDP	no data	leg dystonia, generalized within 5 years, parkinsonism features after 16 years with dystonia	49 y	1 sister	[71]
heterozygous XDP	missense mutation DSC3 change (C>T) X-chromosome monosomy in a subset of cells	abnormal putamen and caudate, leg and hand dystonia, then parkinsonism with some dystonia Turner syndrome (short stature, drooping ears)	50 y	3 brothers, maternal grandfather, mother (?)	[71,73]
heterozygous XDP	3 XDP-specific mutant alleles at DXS8030, DXS8101, and DXS559	parkinsonism: shuffling gait, breakdown of limb RAMs, mild retropulsion leg chorea that generalized	51 y	maternal grandfather, 1 brother, 1 sister, 1 daughter	[113]
heterozygous XDP	5 disease-specific single-nucleotide changes 1 48 bp deletion 1 SVA retrotransposon insertion skewed X-inactivation (98:2%), only C>T expressed (no other changes)	parkinson symptoms caudate atrophy	57 y		[114]
XDP	no data	left foot dystonia	59 y		[113]
homozygous XDP	3 XDP-specific mutant alleles at DXS8030, DXS8101, and DXS559	parkinsonism: stooped posture, breakdown of RAMs of limbs, shuffling gait, retropulsion chorea in arm	72 y	2 sons	[74]
heterozygous XDP	3 XDP-specific mutant alleles at DXS8030, DXS8101, and DXS559	parkinsonism: shuffling gait, breakdown of limb RAMs, hypomimia, stooped posture, micrographia, absent arm swing bilaterally, tremor effects	75 y	1 daughter, 3 sons	[74]
heterozygous XDP	3 XDP-specific mutant alleles at DXS8030, DXS8101, and DXS559	parkinsonism: breakdown of RAMs, shuffling gait	75 y	1 daughter, 4 sons	[74]
heterozygous XDP	disease-specific haplotype SVA retrotransposon insertions	no neurological defects	NA	1 brother	[115]
heterozygous XDP	disease-specific haplotype SVA retrotransposon insertions	no neurological defects	NA	1 brother	[115]
heterozygous *TAF1* mut	missense mutation (c.745G > A; p.Gly249Arg) skewed X-inactivation (95:5)	global developmental delay, delayed psychomotor development	12 mo	1 brother, mother, paternal grandmother with unidentified psychiatric pathology	[62]
heterozygous *TAF1* mut	missense mutation (c.1786C > T; p.Pro596Ser)	no data	NA	3 sons	[3]
heterozygous *de novo TAF1* mut	missense mutation (c.2039G > A p.Gly680Asp) skewed X-inactivation (> 90:10%)	dysmorphism in the face, hands, feet and neck, global developmental delay cardiovascular issues (heart murmur, pulmonary artery atresia and hypoplasia)	birth	none	[58]
*de novo TAF1* mut	missense mutation (c.2039G > A; p.Gly680Asp)	congenital heart disease (pulmonary artery atresia)		none	[56]
*TAF1* mut	missense mutation (g.23335G >T; exon18: c.2774G > A:p.G925D) assumed de novo (not confirmed)	at birth: jaundice, microcephaly, gastroesophageal reflux, hypotonia and congenital cardiopathy. during childhood: delayed developmental milestones, fine motor incoordination, delayed speech, moderate intellectual disability. Asthma, pneumonia dysmorphic features	birth	none confirmed	[116]
*de novo TAF1* mut	missense mutation (c.2933C > T; p.Thr978Met)	congenital heart disease (Ebstein, ventricular septal defect (VSD), hypoplastic aortic arch)		none	[56]
heterozygous *de novo TAF1* mut	missense mutation (c.3035C > T p.Thr1012Ile) skewed X-inactivation	learning disability, natal oral cleft, ventricular septal defect, swirling pigmentary disturbances, postnatal growth retardation, global developmental delay, generalized hypotonia, hypertelorism and other facial developmental abnormalities, sensorineural hearing impairment	birth	none	[58]
*de novo TAF1* mut	missense mutation (c.3035C > T; pThr1012Ile)	congenital heart disease (VSD, PA stenosis)		none	[56]
heterozygous *TAF1* mut	missense mutation (c.3568C > T; p.Arg1190Cys)	no symptoms	NA	2 sons	[117]
heterozygous *TAF1* mut	frameshift mutation (c.3708A > G; p.(arg1228Ilefs*16) alternative transcript (splice site) with 28 bp deletion, resulting in a frameshift and a premature stop codon	no symptoms	NA	1 son	[3]
heterozygous *TAF1* mut	missense mutation (c.4010T>C) skewed X-inactivation (99:1%)	no symptoms (healthy)	NA	2 sons	[3]

Common features among affected females with non-XDP *TAF1* mutations include intellectual disability and facial abnormalities [58], as well as congenital heart disease in a similar manner to their male counterparts ([56,116]; table 2). However, the proportion of affected versus healthy females with *TAF1* mutations is difficult to estimate because many female cases without symptoms, or with mild presentations, may have escaped identification. In females with *TAF1* mutations, missense mutations are most common, although a frameshift mutation in *TAF1* has also been identified [3]. As in XDP, skewed X-inactivation of *TAF1* has been reported in females carrying *TAF1* mutations, which presented with a spectrum of clinical symptoms or were asymptomatic, while they had affected sons [3,57,58]. Thus, X-inactivation status in heterozygous female carriers of XDP or other *TAF1* mutations may contribute to the highly heterogeneous clinical features.

### 5.2. Functional compensation of TAF1 activity

In conditions of reduced *TAF1* expression, it is intriguing to speculate that a paralogous gene may compensate for TAF1 activity. *TAF1L* is a human paralogue of TAF1 that is located on chromosome 9 at an intronless locus (9p21.1) (Ensembl: ENSG00000122728). *TAF1L* probably arose from a retrotransposon gene insertion and can be translated into an intact protein (Uniprot: 1826 aa, Q8IZX4; Antonova S, Timmers HTM, unpublished results [2024]), which shows approximately 95% amino acid homology with TAF1 [119]. *TAF1L* has just one transcript (6216 bp) and is composed of the 38 canonical *TAF1* exons including exon 35′ (excluding exons d_1_– d_5_) [32].

TAF1L has been proposed to compensate for TAF1 in spermatocytes, where TAF1L is exclusively expressed [119]. In these meiotic cells, autosomes are transcriptionally active, but X and Y chromosomes are silenced owing to their condensation into heterochromatin in a transcriptionally inactive compartment. Therefore, autosomal genes may functionally substitute activities of genes on the sex chromosomes during this period [120], such as initiation of RNA pol II-dependent transcription by TAF1L-containing TFIID in spermatocytes replacing TAF1. TAF1L is also dysregulated in a number of cancers, indicating that it may have a similar function to TAF1 in the regulation of tumorigenesis [109,121].

### 5.3. Tissue-specific roles of TAF1

As highlighted above, *TAF1* and its isoforms may be differentially expressed between cell types and tissues. TAFs, including TAF1, have activator-dependent transcription functions, which are both promoter- and tissue-specific [122]. For example, while TAF4B is expressed in many different tissues, deletion of this gene results in viable but infertile offspring owing to its requirement for folliculogenesis [123]. Additionally, *TAF1* may have a specific role in cardiac function, where loss of function causes congenital heart disease, owing to the epigenetic modulatory role of its double BrD in regulating foetal myosins [49]. In this manner, TAF1 could potentially be involved in the repression of the atrial-specific SMyHC3 promoter leading to a cardiac-specific effect of its variants, or alternatively just results as a secondary tissue to be affected by the TAF1 dysfunction.

Cell type-specific differences in TAF1 expression could potentially differentially influence tissues affected by pathology such as the brain and specific stages of development and in neurodegenerative states. For example, the promoter of the sense transcript of *TAF1*–MTS exons d_2_, d_3_ and d_4_ has been described as under the control of an *Ikaros* element, which is specifically expressed in the striatum of the developing brain [32]. Furthermore*,* the most commonly known driver of microexon inclusion (e.g. 34′) is neuron-specific splicing factor SRRM4, which incorporates brain-specific microexons into *TAF1* mRNAs [36,124]. Finally, the *TAF1*−32i variant, that is elevated in XDP, is primarily in dividing cells rather than differentiated neurons [4], indicating a role for this variant in specific cell types. For example, *TAF1*−32i was higher in XDP fibroblasts, iPSCs and NSCs, with low levels in cortical neurons, GABAergic neurons, and NSC-derived neurons compared with control cells [4]. Higher *TAF1*−32i levels were also reported in XDP fibroblasts and iPSCs compared with blood [35], indicating tissue-specific levels of this transcript.

Whether TAF1 expression, splicing and isoform abundance changes with ageing is not known yet. Diseases of TAF1 dysfunction such as XDP and XLID predominantly affect the brain, and postnatal depletion of *Taf1* in rodents takes several months to induce a phenotype of neurocognitive decline [38] indicating that TAF1 dysfunction plays a larger role with increasing age or that symptoms have delayed manifestation. Elucidation of the age-specific expression of TAF1 will be key to targeting TAF1-induced diseases.

## 6. Conclusions and further remarks

In conclusion, TAF1 has multiple functions governed by its activity and interacting partners that ultimately regulate RNA pol II-dependent transcription of many genes. *TAF1* is a key gene for development and complete deletion results in embryonic lethality in zebrafish and probably humans and other animals (https://gnomad.broadinstitute.org/gene/ENSG00000147133?dataset=gnomad_r4). Congenital hypomorphic mutations in *TAF1* in humans result in aberrant development of the brain with corresponding intellectual disability and can also cause congenital heart disease. *TAF1* dysregulation in XDP results in striatal neurodegeneration and consequential loss of motility in middle age, similar to that in HD and PD. However, while much is known about the *TAF1* gene and its protein structure and function, and diseases involving TAF1 dysregulation have been identified, there is still much to be uncovered to mechanistically connect this gene with disease causation.

We have provided various examples of how *TAF1* variant expression may be linked to the neurodegenerative condition XDP, such as increased levels of *TAF1*−32i. However, the cell type-specificity of these variants and their abundance in various tissues has not been comprehensively explored. Tissue-specific splicing factors may be key to uncovering the regulation of *TAF1* transcripts and their relationship to pathogenesis, such as why the striatum is predominantly affected in XDP, with application to HD and other motor disorders.

Functional aspects of TAF1 biology may play essential roles in pathogenesis. We highlight that *TAF1* is primarily expressed in dividing rather than differentiated cells. TAF1 has also been linked to tumorigenesis, and we speculate it might be differentially regulated by cancer cells as evidenced in multiple types of cancers. This hypothesis would make sense given that the TFIID complex regulates basal transcription, which is required for cell growth. However, how different mutations affect the function of TAF1 in disease is not yet clear.

Genetic animal models are important for understanding systems biology and stages of pathogenesis, particularly for neurodegenerative and other progressive conditions. While *TAF1* KO models have been developed in organisms such as for zebrafish, embryonic lethality in these studies highlights the key role of TAF1 function in development but these models do not allow the investigation of *TAF1* loss of function effects in full KOs. Additionally, rodent studies with postnatal *Taf1* KD support the role of *TAF1* loss of function in motor decline, while the relevant molecular and cellular mechanisms remain to be elucidated. Modelling of XDP is a challenge because the sequence of intron 32 is not conserved between mice and humans. Thus, humanizing the mouse *Taf1* gene to introduce the SVA into mice will be necessary to effectively model this disease in animals. In the last 10 years, the body of research describing the role of *TAF1* in XDP has increased exponentially, but new techniques will be required to elucidate the mechanistic role of this gene in this complex disease.

## Data Availability

This article has no additional data.

## Footnotes

^1^

*cTAF1*, canonical human *TAF1* transcript; n*TAF1*, neuronal human *TAF1* transcript; *cTaf1*, canonical mouse *Taf1* transcript; *nTaf1*, neuronal mouse *Taf1* transcript; C-TAF1, canonical protein TAF1 isoform; N-TAF1, neuronal protein TAF1 isoform.
